# Subgroup analysis of treatment pathways and clinical outcomes in Hodgkin lymphoma in Latin America from the retrospective B-HOLISTIC study

**DOI:** 10.1038/s41598-025-07704-0

**Published:** 2025-07-07

**Authors:** Alvaro Hernandez-Caballero, Ruben Salazar, Marta Zerga, Silvia Rivas-Vera, Zhongwen Huang, Amado Karduss

**Affiliations:** 1 Unidad Medica de Alta Hospital de Especíalídades Dr. Antonio Fraga Mouret Centro Medico Nacional la Raza, Mexico City, Mexico; 2 Clínica de Oncología Astorga, Medellín, Colombia; 3 Instituto de Oncología Angel H. Roffo, Buenos Aires, Argentina; 4 Department of Hematology, Instituto Nacional de Cancerología México, Mexico City, Mexico; 5 Takeda R&D Data Science Institute, Takeda Pharmaceuticals International Co., Cambridge, USA; 6 Instituto de Cancerología Las Americas AUNA, Medellín, Colombia

**Keywords:** Real-world study, Hodgkin lymphoma, Relapsed/refractory, Latin America, Treatment outcomes, Hodgkin lymphoma, Cancer therapy

## Abstract

Real-world data on Hodgkin lymphoma (HL) treatment and outcomes in Latin America are limited. The B-HOLISTIC study (2010–2013) retrospectively assessed treatment patterns and outcomes in patients with stage IIB–IV classical HL receiving frontline chemotherapy (frontline cHL) or relapsed/refractory HL (RRHL) in regions outside Europe and North America. This subgroup analysis presents findings from patients enrolled from treatment centers in Argentina, Colombia, and Mexico. The primary endpoint was progression-free survival (PFS) in patients with RRHL. Other endpoints included overall survival (OS), best clinical response, and treatment patterns. In Latin America (frontline cHL: 344; RRHL: 92), the most common frontline regimen was ABVD (doxorubicin, bleomycin, vinblastine, dacarbazine). In patients with RRHL receiving salvage chemotherapy (92.4%), common regimens were ESHAP (etoposide, methylprednisolone, cytarabine, cisplatin) and ICE (ifosfamide, carboplatin, etoposide). Overall, 68.2% of eligible patients with RRHL underwent stem cell transplantation (SCT). Median PFS and OS in the RRHL group was 20.2 and 87 months, respectively; the 5-year PFS rate was 27.1%. Survival outcomes were improved in patients who underwent SCT than those without SCT. Median PFS and OS in the cHL group were not reached. These data demonstrated that while real-world HL treatment in Latin America was consistent with guidelines during the study period, clinical outcomes were suboptimal compared with developed regions. This emphasizes the need for novel therapies and improved progression to SCT in this patient population. These findings may offer valuable insights and serve as a reference for future studies in this region. ClinicalTrials.gov Identifier: NCT03327571, registered October 31, 2017.

**Supplementary Information:**

The online version contains supplementary material available at 10.1038/s41598-025-07704-0.

## Introduction

Continuous advancements in diagnostic and treatment approaches in Hodgkin lymphoma (HL) in the past 20 years have increased the cure rates in classical HL (cHL) with a 5-year overall survival (OS) rate of 80–90% in most developed countries^1–3^. Despite this, 10–30% of patients have relapsed/refractory HL (RRHL)^2,3^. In patients with RRHL, second-line treatment with intensive salvage chemotherapy followed by stem cell transplantation (SCT) for eligible patients is the standard of care^1–5^ and has shown improved patient survival rates (40–60%)^6–9^. The use of novel targeted salvage therapies with SCT has been shown to improve clinical outcomes in patients with RRHL^1,3,10,11^; however, there are many challenges with their access in several low- and middle-income countries (LMICs).

In low- and middle-income regions like Latin America, HL affects approximately 5000 new patients annually^12^. In Latin America, limited access to healthcare and potentially life-saving chemotherapy regimens may result in lower cure rates for HL than in developed countries^13–15^. Additionally, the utilization of new therapies and the applicability of evidence-based guidelines from Europe and North America in this region are unknown. While national registries such as Brazilian Hodgkin Lymphoma Registry (including data from 1507 patients)^16,17^ and local reports^15,18^ provide valuable insights into treatment patterns and outcomes, comprehensive regional data, particularly from Latin America, remain limited. Efforts from LMICs, such as a multi-institutional study in India^19^, help shed light on HL in resource-constrained settings. However, the findings from these studies may not be directly applicable to Latin America due to differences in healthcare infrastructure and treatment access. Overall, there is a notable scarcity of data on treatment patterns and clinical outcomes for HL in LMICs, especially in Latin America. Recently, there has been a considerable increase in the amount of research related to HL in Latin America; however, most of these contributions are either epidemiological studies^13,20^ or are limited by small sample sizes and single-country or single-center experiences^14,15,21^.

Real-world data on treatment patterns and clinical outcomes in Latin America are important due to reported disparities in clinical presentation and outcomes based on various factors such as age, sex, race, ethnicity, geography, socioeconomic condition, and country development level^22–24^. These data could help make informed decisions about patient care and health policy in this region and in developing strategies to improve diagnosis and treatment at national and regional levels.

The B-CD30+ HOdgkin Lymphoma International Multi-Center Retrospective Study of Treatment PractIces and OutComes (B-HOLISTIC) study described real-world treatment pathways and clinical outcomes in a large cohort of patients with cHL and RRHL from 12 countries outside Europe and North America, including three countries from Latin America. This large-scale study reported that although treatment practices in these countries were in accordance with guideline recommendations at the time of the study, the clinical outcomes in these regions were suboptimal compared with reports from Europe and North America^25^. This article describes the real-world treatment patterns and clinical outcomes in HL from the Latin America subgroup (Argentina, Colombia, and Mexico) of the B-HOLISTIC study.

## Methods

### Study design and patients

The B-HOLISTIC study (NCT03327571) was an international, multicenter, retrospective, observational study of patients with advanced stage IIB–IV cHL who had received frontline chemotherapy treatment (frontline cHL) with or without radiotherapy (RT), and/or patients with a diagnosis of RRHL^25^. This subgroup analysis was subsequently conducted to further investigate treatment patterns and clinical outcomes specifically within Latin America. For this subgroup analysis, patients who provided written informed consent were enrolled from highly specialized treatment centers in Argentina (8 centers), Colombia (8 centers), and Mexico (3 centers; Supplementary material S1). Data were collected at participating centers, between January 1, 2010 and December 31, 2013, and patients were followed from diagnosis until death or last follow-up (whichever occurred first before March 4, 2020). Patients initially diagnosed with cHL who progressed to RRHL during the study period were included in both cHL and RRHL groups for analyses. Details on the selection of patients and participating treatment centers, the minimum data set required for each patient, and data collection methods have been described previously^25^. The study was conducted according to the Declaration of Helsinki and the International Conference on Harmonization Guidelines on Good Clinical Practice and was approved by the relevant Independent Ethics Committee/Institutional Review Boards at each center (Argentina: Hospital Italiano de Buenos Aires Research Protocols Ethics Committee, Instituto de Oncología Ángel H. Roffo Research Ethics Committee, Clinical Research Ethics Committee, Institutional Committee on Health Research Ethics Hospital Privado Centro Medico de Cordoba, Hospital Universitario Austral Institutional Review Board, Hospital Británico Institutional Review Committee, Instituto Medico Especializado Alexander Fleming Research Ethics Committee, Centro de Investigación y Prevención Cardiovascular Clinical Research Ethics Committee; Colombia: Instituto de Cancerología Ethics Committee, HSJ-FUCS Human Research Ethics Committee, HPTU Research and Ethics Committee, Fundación Hospitalaria San Vicente Paúl Research Ethics Committee, Clínica De Oncología Astorga Ethics Committee, Fundación Oftalmológica de Santander [FOSCAL] Research and Ethics Committee, Ethics Committee of the Hospital Universitario Mayor Mederi, Clinica de la Fundación Cardioinfantil Research Ethics Committee; Mexico: Centro de Investigacíon Farmacéutica Especializada de Occidente Research Ethics Committee, Ethics Committee Centro Medico Nacional Research Ethics Committee, Research Ethics Committee of the Antiguo Hospital Civil de Guadalajara Fray Antonio Alcalde).

### Study outcomes

The variables related to treatment and imaging patterns, chemotherapy regimens, clinical outcomes, and adverse events (AEs) assessed in the B-HOLISTIC study have been previously described^25^. The primary endpoint was PFS in patients with RRHL, defined as the time from the initiation of first salvage treatment for RRHL to the first documentation of relapse or disease progression, or death. Secondary endpoints included treatment patterns in both groups, PFS in the frontline cHL group, overall survival (OS) in both groups, best clinical response (i.e. complete remission [CR], partial remission [PR], stable disease [SD], or progressive disease [PD]) to frontline or first salvage treatment, time to response (CR or PR), SCT patterns and associated clinical outcomes, and AEs. The term ‘SCT’ is used to refer to all patients who underwent SCT, irrespective of the type of SCT.

### Statistical analysis

All analyses were performed separately for the frontline cHL and RRHL groups for all patients with available data. No imputation was done where data were missing. Patient and disease characteristics, treatment patterns, and AEs were presented as descriptive statistics (median and range or interquartile range for continuous variables and number, percentage for categorical variables) using the number of subjects. PFS and OS were analyzed using the Kaplan–Meier (KM) method. The Statistical Analysis System (SAS®) Software, Version 9.4 (SAS Institute Inc., Cary, NC, USA) was used for all analyses. A detailed description of the B-HOLISTIC statistical analyses has been described previously^25^.

## Results

### Patient disposition

**Fig. 1:**
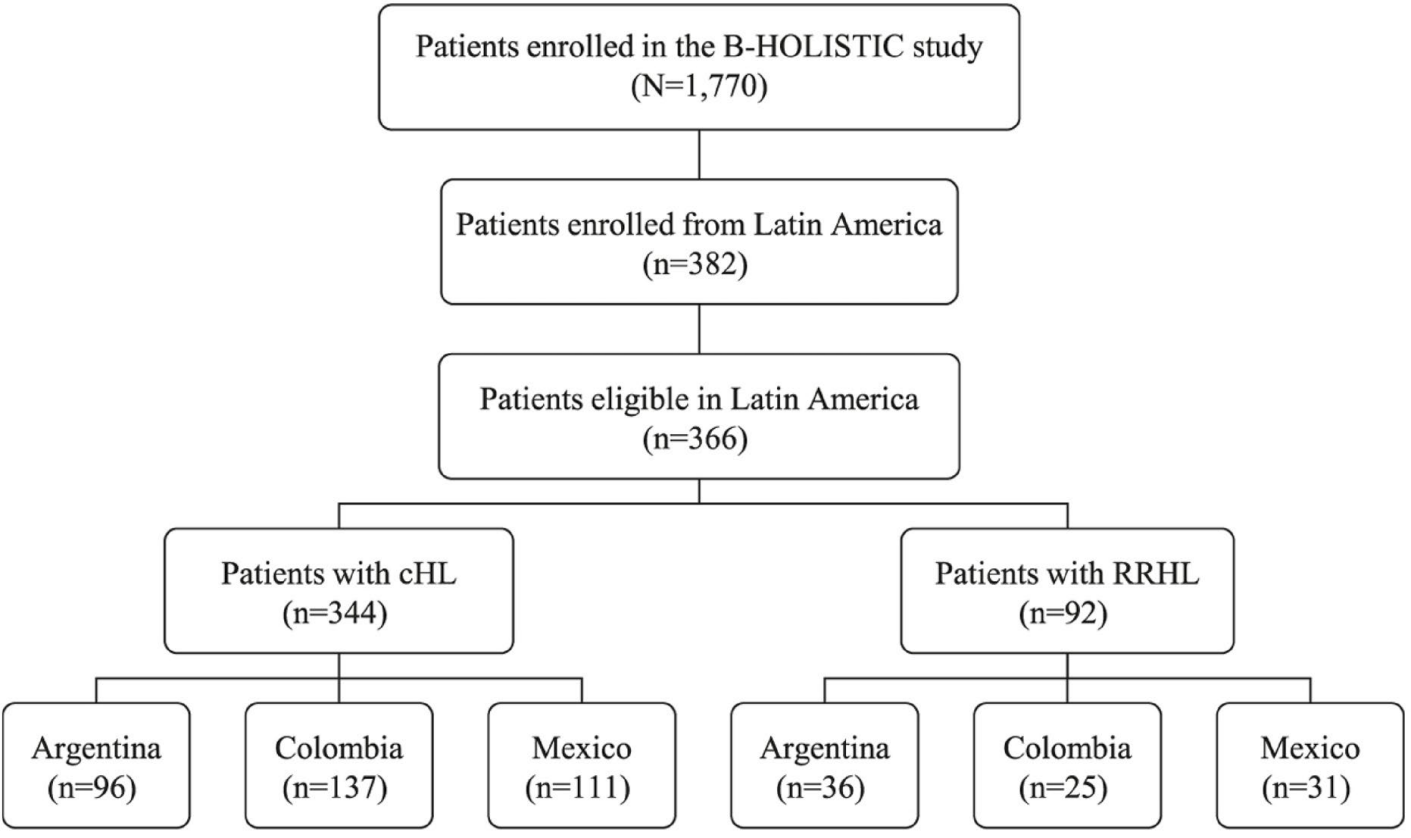
Patient disposition. *cHL* classical Hodgkin lymphoma, *RRHL* relapsed/refractory Hodgkin lymphoma. *Note*: 70 patients with an initial diagnosis of cHL who progressed to RRHL during the study period were included in both frontline cHL and RRHL groups for analysis. The flowchart depicts the distribution and eligibility of patients enrolled in the B-HOLISTIC study LATAM subgroup analysis. 1770 patients were enrolled in B-HOLISTIC, with 382 being from Latin America. Among these, 366 patients were eligible for inclusion in this subanalysis. The eligible Latin American patients were further divided into two groups: those with classical Hodgkin Lymphoma (cHL), and those with relapsed/refractory Hodgkin lymphoma (RRHL). The cHL group included 344 patients, with the following country breakdown: Argentina: 96 patients; Colombia: 137 patients; Mexico: 111 patients. The RRHL group comprised 92 patients, distributed as follows: Argentina: 36 patients; Colombia: 25 patients; Mexico: 31 patients.

A total of 366 patients (Argentina: 106, Colombia: 145, and Mexico: 115) were enrolled in Latin America (Fig. 1). Of these, 344 patients were analyzed in the cHL group and 92 patients in the RRHL group. The median (range) study follow-up in the frontline cHL group was 58.8 (3.9–104.2), 56.4 (0.2–116.4), and 60 (0.4–114.0) months in Argentina, Colombia, and Mexico, respectively. In the RRHL group, the median (range) study follow-up was 45.6 (0.8–90.2), 37.2 (0.03–108.0), and 36 (0.6–123.2) months in Argentina, Colombia, and Mexico, respectively.

### Patients with RRHL

#### Patient demographics and baseline characteristics

The median age in the RRHL group was 35.0 years (Table 1), with a similar proportion of males and females (52% vs. 48%). Most patients (41.3%) were of an ethnicity other than those listed. At relapse/refractory diagnosis, most patients (26.1%) were at stage III-B (Ann Arbor). Compared with Argentina, a higher proportion of patients in Mexico and Colombia had B symptoms (33% vs. 68% and 68%, respectively) at relapse/refractory diagnosis. Additionally, patients in Colombia had a higher rate of extranodal involvement than patients in Mexico or Argentina, but no patients in Colombia had bulky disease. Refractory disease was more common in patients from Argentina and Mexico (44% and 58%, respectively), while relapse disease was more common in patients from Colombia (56%).

**Table 1:** Baseline patient characteristics in patients with RRHL in Latin America: overall and by country.

Characteristic	Overall (*n* = 92)^a^	Argentina (*n* = 36)	Colombia (*n* = 25)	Mexico (*n* = 31)
Median age, years (range)	35.0 (18.0–85.0)	35.5 (20.0–85.0)	41.0 (18.0–77.0)	32.0 (90.0–71.0)
< 60, *n* (%)	81 (88.0)	32 (88.9)	22 (88.0)	27 (87.1)
≥ 60, *n* (%)	11 (12.0)	4 (11.1)	3 (12.0)	4 (12.9)
Male, *n* (%)	48 (52.2)	23 (63.9)	12 (48.0)	13 (41.9)
Ethnicity/race, *n* (%)				
White/Caucasian	28 (30.4)	26 (72.2)	1 (4.0)	1 (3.2)
Black or African American	2 (2.2)	1 (2.8)	1 (4.0)	0
American Indian or Alaska Native	2 (2.2)	1 (2.8)	0	1 (3.2)
Native Hawaiian or another Pacific Islander	0	0	0	0
Not reported	22 (23.9)	6 (16.7)	13 (52.0)	3 (9.7)
Other	38 (41.3)	2 (5.6)	10 (40.0)	26 (83.9)
Ann Arbor stage at first diagnosis, *n* (%)				
IA–IIA	15 (16.3)	11 (30.6)	3 (12.0)	1 (3.2)
IIB	15 (16.3)	5 (13.9)	5 (20.0)	5 (16.1)
IIIA	13 (14.1)	9 (25.0)	0 (0.0)	4 (12.9)
IIIB	24 (26.1)	4 (11.1)	7 (28.0)	13 (41.9)
IVA	6 (6.5)	4 (11.1)	1 (4.0)	1 (3.2)
IVB	10 (10.9)	3 (8.3)	5 (20.0)	2 (6.5)
Unknown	9 (9.8)	0 (0.0)	4 (16.0)	5 (16.1)
PET/PET-CT at baseline, *n* (%)^c^	11 (21.2)	11 (36.7)	0 (0.0)	0 (0.0)
Presence of B symptoms, *n* (%)	50 (54.3)	12 (33.3)	17 (68.0)	21 (67.7)
Extranodal involvement at diagnosis, *n* (%)^d^	21 (23.3)	8 (22.2)	7 (30.4)	6 (19.4)
Bulky disease ≥ 5 cm at diagnosis, *n* (%)^d^	18 (20.0)	11 (30.6)	0 (0.0)	7 (22.6)
ECOG PS, *n* (%)	*n* = 52	*n* = 22	*n* = 13	*n* = 17
0	33 (63.5)	15 (68.2)	5 (38.5)	13 (76.5)
1	16 (30.8)	6 (27.3)	7 (53.8)	3 (17.6)
2	1 (1.9)	0 (0.0)	0 (0.0)	1 (5.9)
3	2 (3.8)	1 (4.5)	1 (7.7)	0 (0.0)
4	0 (0.0)	0 (0.0)	0 (0.0)	0 (0.0)
Unknown^b^	40	14	12	14
cHL histological subtype, *n* (%)				
Nodular sclerosis	69 (75.0)	17 (47.2)	11 (44.0)	9 (29.0)
Mixed cellularity	12 (13.0)	2 (5.6)	0 (0.0)	3 (9.7)
Lymphocyte-depleted	1 (1.1)	0 (0.0)	0 (0.0)	0 (0.0)
Lymphocyte-rich	1 (1.1)	1 (2.8)	0 (0.0)	0 (0.0)
Unknown^b^	9 (9.8)	0 (0.0)	4 (16.0)	1 (3.2)
Josting score^e^, *n* (%)	*n* = 35	*n* = 19	*n* = 6	*n* = 10
0	8 (22.9)	6 (31.6)	1 (16.7)	1 (10.0)
1	13 (37.1)	5 (26.3)	3 (50.0)	5 (50.0)
2	11 (31.4)	8 (42.1)	2 (33.3)	1 (10.0)
3	3 (8.6)	0 (0.0)	0 (0.0)	3 (30.0)
Unknown	13	1	9	3

*cHL* classical Hodgkin lymphoma, *CT* computed tomography, *ECOG PS* Eastern Cooperative Oncology Group Performance Status, *PET* positron emission tomography, *RRHL* relapsed/refractory Hodgkin lymphoma.

^a^70 patients with an initial diagnosis of cHL progressed to RRHL during the study period and were included in both cHL and RRHL groups.

^b^Unknown: there is no entry in the medical record or patient chart relating to an item as confirmed by the site.

^c^PET-CT data at baseline was only available for some patients and, therefore, the total ‘*n*’ values for these data were: Overall = 52, Argentina = 30, Colombia = 17, and Mexico = 5.

^d^Data on extranodal involvement and bulky disease was available for only following number of patients: Overall = 90, Argentina = 36, Colombia = 23, and Mexico = 31.

^e^Josting score parameters are defined as time to relapse ≤ 12 months, clinical stage III or IV at relapse, and anemia (hemoglobin < 10.5 g/dL for females and < 12 g/dL for males) at relapse.

#### Treatment patterns

In the RRHL group, 92.4% of patients received intensive first salvage chemotherapy (Table 2). The most commonly used salvage regimens were etoposide, methylprednisolone, cytarabine, cisplatin (ESHAP) in Argentina (40.0%), ifosfamide, carboplatin, etoposide (ICE) in Colombia (63.6%), and etoposide, ifosfamide, mesna, mitoxantrone (MINE) in Mexico (32.1%). Doxorubicin, bleomycin, vinblastine, dacarbazine (ABVD) was used as salvage regimen in four patients; of these, one had received cyclophosphamide, doxorubicin, vincristine, and prednisone (CHOP) as frontline treatment, while the other three had previously been treated with ABVD. Radiotherapy was administered at relapse/refractory diagnosis in 25% of patients with RRHL in Latin America, most commonly with chemotherapy (94%) for a radical/cure intent. PET/PET-CT scans were performed at the time of documented relapse and/or refractory disease in 44.2% of RRHL patients.

**Table 2:** Treatment patterns in patients with RRHL in Latin America: overall and by country.

	Overall (*n* = 92)^a^	Argentina (*n* = 36)	Colombia (*n* = 25)	Mexico (*n* = 31)
First salvage chemotherapy^b^, *n* (%)	85 (92.4)	35 (97.2)	22 (88.0)	28 (90.3)
ABVD	4 (4.7)	0 (0.0)	2 (9.1)	2 (7.1)
DHAP	4 (4.7)	2 (5.7)	2 (9.1)	0 (0.0)
ESHAP	16 (18.8)	14 (40.0)	1 (4.5)	1 (3.6)
GCD	1 (1.2)	0 (0.0)	0 (0.0)	1 (3.6)
ICE	18 (21.2)	3 (8.6)	14 (63.6)	1 (3.6)
IGEV	10 (11.8)	9 (25.7)	1 (4.5)	0 (0.0)
MINE	10 (11.8)	1 (2.9)	0 (0.0)	9 (32.1)
Rituximab	1 (1.2)	0 (0.0)	0 (0.0)	1 (3.6)
BEACOPP	9 (10.6)	6 (17.1)	0 (0.0)	3 (10.7)
ABVD followed by escalated BEACOPP	4 (4.7)	0 (0.0)	0 (0.0)	4 (14.3)
Other	9 (10.6)	1 (2.9)	2 (9.1)	6 (21.4)
Number of chemotherapy regimens, median (range)	2.0 (1.0–5.0)	2.0 (1.0–5.0)	1.5 (1.0–3.0)	2.0 (1.0–4.0)
Patients receiving PET or PET-CT scans, *n* (%)	*n* = 52 (56.5%)	*n* = 30 (83.3%)	*n* = 17 (68.0%)	*n* = 5 (16.1%)
Number of PET or PET-CT scans, median (range)	2.0 (1.0–13.0)	2.0 (1.0–13.0)	1.0 (1.0–5.0)	2.0 (1.0–3.0)
Patients receiving CT scans, *n* (%)	*n* = 80 (87.0%)	*n* = 29 (80.6%)	*n* = 23 (92.0%)	*n* = 28 (90.3%)
Number of CT scans, median (range)	3.0 (1.0–8.0)	2.0 (1.0–8.0)	3.0 (1.0–7.0)	3.0 (1.0–6.0)
Radiotherapy at relapse/refractory diagnosis, *n* (%)	23 (25.0)	6 (16.7)	5 (20.0)	12 (38.7)
Median no. radiotherapy treatments, *n* (range)	1.0 (1.0–1.0)	1.0 (1.0–1.0)	1.0 (1.0–1.0)	1.0 (1.0–1.0)
Patients eligible for SCT, n (%)	66 (71.7)	33 (91.7)	17 (68.0)	16 (51.6)
Reason for SCT ineligibility, *n* (%)	*n* = 26	*n* = 3	*n* = 8	*n* = 15
Advanced age	2 (7.7)	1 (33.3)	1 (12.5)	0 (0.0)
Comorbid conditions	3 (11.5)	0 (0.0)	2 (25.0)	1 (6.7)
Chemoresistant disease	1 (3.8)	0 (0.0)	0 (0.0)	1 (6.7)
Cumulative toxicities	1 (3.8)	0 (0.0)	0 (0.0)	1 (6.7)
Patient refusal	0 (0.0)	0 (0.0)	0 (0.0)	0 (0.0)
Inability to mobilize stem cells	1 (3.8)	0 (0.0)	1 (12.5)	0 (0.0)
Loss of response to chemotherapy	1 (3.8)	0 (0.0)	0 (0.0)	1 (6.7)
Other	3 (11.5)	0 (0.0)	2 (25.0)	1 (6.7)
Unknown	14 (53.8)	2 (66.7)	2 (25.0)	10 (66.7)
Patients undergoing SCT, *n* (%)	45 (68.2)	28 (84.8)	10 (58.8)	7 (43.8)
Reason for not undergoing SCT, *n* (%)	*n* = 21	*n* = 5	*n* = 7	*n* = 9
Advanced age	0 (0.0)	0 (0.0)	0 (0.0)	0 (0.0)
Comorbid conditions	2 (9.5)	0 (0.0)	2 (28.6)	0 (0.0)
Cumulative toxicities	0 (0.0)	0 (0.0)	0 (0.0)	0 (0.0)
Patient refusal	2 (9.5)	0 (0.0)	1 (14.3)	1 (11.1)
Inability to mobilize stem cells	0 (0.0)	0 (0.0)	0 (0.0)	0 (0.0)
Loss of response to chemotherapy	6 (28.6)	0 (0.0)	0 (0.0)	6 (66.7)
Financial reasons	0 (0.0)	0 (0.0)	0 (0.0)	0 (0.0)
Other	8 (38.1)	4 (80.0)	3 (42.9)	1 (11.1)
Unknown	3 (14.3)	1 (20.0)	1 (14.3)	1 (11.1)
Type of SCT, *n* (%)	*n* = 45	*n* = 28	*n* = 10	*n* = 7
ASCT	37 (82.2)	24 (85.7)	8 (80.0)	5 (71.4)
Allo-SCT	3 (6.7)	0 (0.0)	1 (10.0)	2 (28.6)
Both	5 (11.1)	4 (14.3)	1 (10.0)	0 (0.0)
Chemotherapy conditioning regimen for ASCT, *n* (%)	*n* = 44	*n* = 28	*n* = 11	*n* = 5
BEAM	13 (29.5)	9 (32.1)	4 (36.4)	0 (0.0)
CBV	17 (38.6)	17 (60.7)	0 (0.0)	0 (0.0)
BeEAM (bendamustine)	3 (6.8)	0 (0.0)	3 (27.3)	0 (0.0)
Gemcitabine/Busulfan/Melphalan	0 (0.0)	0 (0.0)	0 (0.0)	0 (0.0)
Other	11 (25.0)	2 (7.1)	4 (36.4)	5 (100.0)
Remission state prior to SCT, *n* (%)	*n* = 46	*n* = 27	*n* = 12	*n* = 7
CR	23 (50.0)	11 (40.7)	6 (50.0)	6 (85.7)
PR	19 (41.3)	15 (55.6)	3 (25.0)	1 (14.3)
SD	4 (8.7)	1 (3.7)	3 (25.0)	0 (0.0)
Non-SCT, *n* (%)	47 (51.1)	8 (22.2)	15 (60.0)	24 (77.4)
Frontline treatment	47 (100.0)	8 (100.0)	15 (100.0)	24 (100.0)
Second-line treatment	41 (87.2)	7 (87.5)	12 (80.0)	22 (91.7)
Third-line treatment	22 (46.8)	4 (50.0)	5 (33.3)	13 (54.2)

*ABVD* doxorubicin bleomycin vinblastine dacarbazine, *Allo-SCT* allogeneic stem cell transplantation, ASCT autologous stem cell transplantation, *BEACOPP* bleomycin etoposide doxorubicin cyclophosphamide vincristine procarbazine prednisone, *BEAM* carmustine etoposide cytarabine melphalan, *BeEAM* bendamustine etoposide cytarabine melphalan, *CBV* cyclophosphamide carmustine etoposide, *CR* complete remission, *DHAP* dexamethasone cytarabine cisplatin, *ESHAP* etoposide methylprednisolone cytarabine cisplatin, *GCD* gemcitabine carboplatine dexamethasone, *ICE* ifosfamide carboplatin etoposide, *IGEV* ifosfamide gemcitabine vinorelbine prednisone, *MINE* etoposide ifosfamide mesna mitoxantrone, *PET-CT* positron emission tomography-computed tomography, *PR* partial remission, *RRHL* relapsed/refractory Hodgkin lymphoma, *SCT* stem cell transplantation, *SD* stable disease.

^a^Seventy patients with an initial diagnosis of frontline cHL progressed to RRHL during the study period and were included in both groups.

^b^In the RRHL group, following relapse or refractory disease diagnosis.

Among the Latin American RRHL cohort, 68.2% (45) of the 66 eligible patients underwent SCT, with the highest proportion in Argentina (84.8%) and the lowest in Mexico (43.8%) (Table 2). The reason for SCT ineligibility was unknown in most cases. In Mexico, the most common reason for eligible patients not undergoing SCT was loss of response to chemotherapy (66.7%), while in Argentina and Colombia, it was not specified. Pre-SCT complete remission rates were 50%, with wide variation across the three countries. Autologous SCT (ASCT) was the preferred modality in most cases (82.2%); 6.7% of patients underwent allogeneic SCT (allo-SCT). Most SCT procedures were performed at clinical stages IIB and IIIA (21.2%). Pre-SCT chemotherapy conditioning regimens varied across the three countries with the most common conditioning regimen being cyclophosphamide, carmustine and etoposide (CBV) in Argentina (60.7%), carmustine, etoposide, cytarabine, melphalan (BEAM) in Colombia (36.4%), and others in Mexico (100%). Post-SCT relapse was reported in 28.9% of patients after a median duration of 14.5 months. Among the patients who relapsed after SCT, the common chemotherapy regimens used were ABVD, ESHAP and gemcitabine, vinorelbine, pegylated liposomal doxorubicin (GVD). Novel targeted therapies were reported to be infrequently prescribed: rituximab was used in one patient as second-line treatment and in another as third-line treatment. Brentuximab vedotin was administered to three patients in third-line settings and two post-SCT. Among patients who did not receive SCT, 87.2% of patients were treated with second-line salvage chemotherapy, with the most common regimen being ICE (17.1%). Approximately half (46.8%) of patients who did not receive SCT had third-line therapy, most commonly with ICE (18.2%).

#### Clinical outcomes

**Fig. 2:**
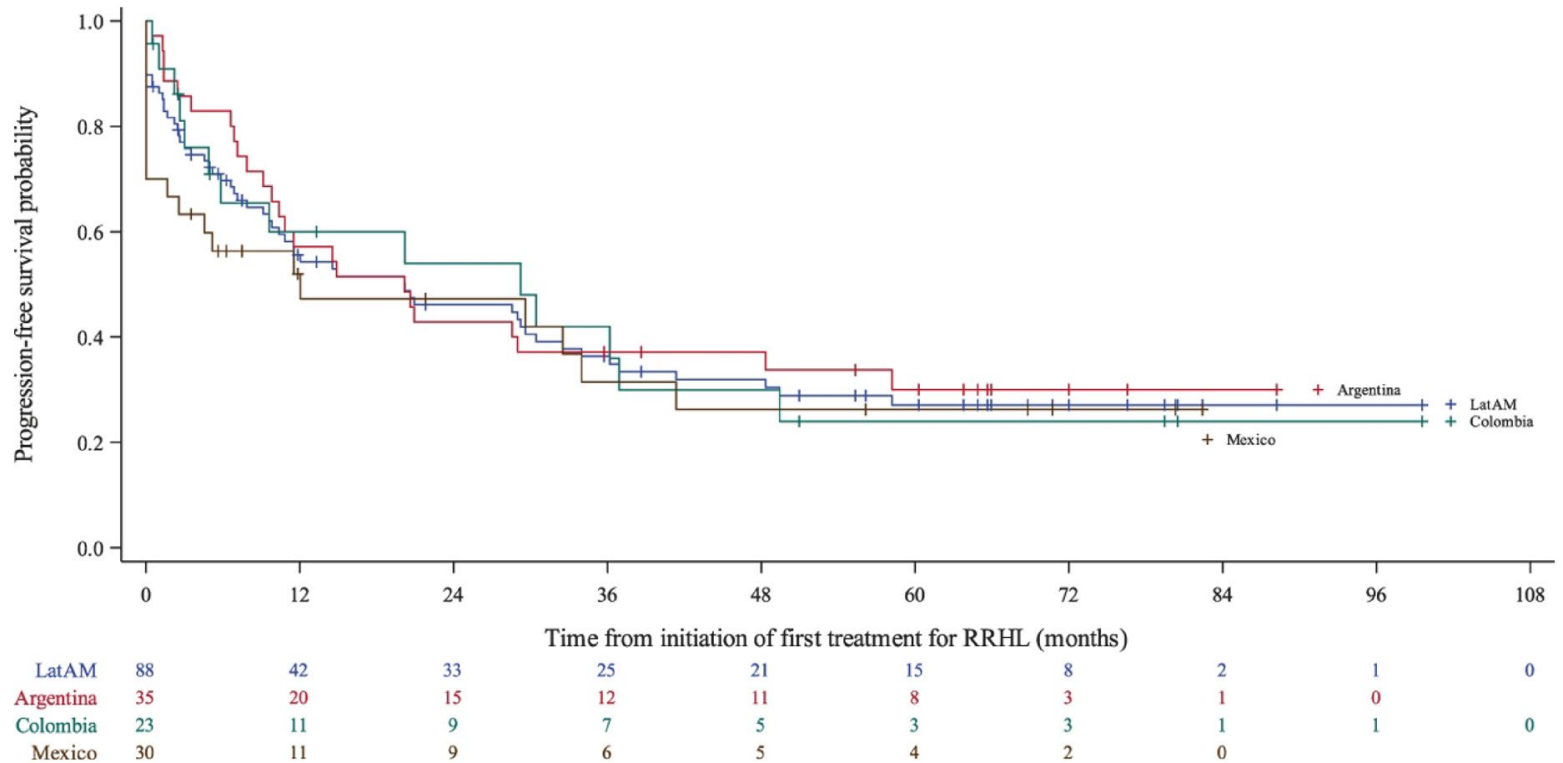
PFS from initiation of first salvage treatment for the RRHL group: overall and by country. *CI* confidence interval, *RRHL* relapsed/refractory Hodgkin lymphoma. *Note*: Four patients with missing data were excluded from the Kaplan–Meier analysis. Median PFS was 20.2 (95% CI 9.8–48.4), 29.2 (95% CI 4.9–49.4), and 12.1 (95% CI 1.7–41.4) months for Argentina, Colombia, and Mexico, respectively. This Kaplan–Meier curve represents the PFS probability over time (in months) for patients from Latin America, broken down by country. The x-axis shows the time from the initiation of the first treatment for RRHL (ranging from 0 to 108 months), while the y-axis shows the progression-free survival probability (ranging from 0.0 to 1.0). The figure highlights differences in survival outcomes across countries, with lines indicating trends in PFS probabilities for each country as well as the overall Latin American cohort. While all countries show a similar downward trend in PFS probability over time, Mexico shows the fastest initial decrease in survival probability.

The median PFS in the RRHL group was 20.2 (95% CI 9.8–30.5) months (Fig. 2 and Table 3). Median PFS was lowest in Mexico (12.1 months) and highest in Colombia (29.2 months). Overall, the 5-year PFS rate was 27.1% (95% CI 17.4–37.7) and was generally similar across the three countries (Colombia: 24%, Mexico: 26%, Argentina: 30%). CR (33.7%) and PR (24.4%) rates to salvage treatment were low. Median OS and the 5-year OS rates after the first relapse were 87.0 (95% CI 59.6–NR) months and 66%, respectively in Latin America, with these parameters being relatively poorer in Mexico compared with the other two countries. Median PFS and OS were better in patients who underwent SCT (29.2 months and NR, respectively) than those without SCT (PFS = 9.6 months and OS = 275.5 months among those receiving ABVD [n = 41] and PFS = 2.5 months and OS = 55.3 months among those receiving bleomycin, etoposide, doxorubicin, cyclophosphamide, vincristine, procarbazine and prednisolone [BEACOPP; n = 9]).

**Table 3:** Clinical outcomes in patients with RRHL in Latin America: overall and by country.

	Overall (*n* = 92)^a^	Argentina (*n* = 36)	Colombia (*n* = 25)	Mexico (*n* = 31)
Median PFS, months (95% CI)	*n* = 88	*n* = 35	*n* = 23	*n* = 30
	20.2 (9.8–30.5)	20.2 (9.8–48.4)	29.2 (4.9–49.4)	12.1 (1.7–41.4)
PFS rate, % (95% CI)				
1-year	55.6 (44.2–65.6)	57.1 (39.3–71.5)	60.0 (35.4–77.8)	52.0 (32.5–68.3)
3-year	36.3 (25.7–47.0)	37.1 (21.7–52.7)	42.0 (19.7–62.9)	31.5 (14.1–50.6)
5-year	27.1 (17.4–37.7)	30.0 (15.6–45.9)	24.0 (7.6–45.3)	26.3 (10.3–45.5)
Best clinical response following first salvage treatment, *n* (%)	*n* = 86	*n* = 35	*n* = 22	*n* = 29
CR	29 (33.7)	10 (28.6)	6 (27.3)	13 (44.8)
PR	21 (24.4)	12 (34.3)	4 (18.2)	5 (17.2)
SD	5 (5.8)	3 (8.6)	0 (0.0)	2 (6.9)
PD	18 (20.9)	6 (17.1)	4 (18.2)	8 (27.6)
Unknown^b^	13 (15.1)	4 (11.4)	8 (36.4)	1 (3.4)
Median time to response (CR, PR), months (range)	*n* = 61	*n* = 26	*n* = 12	*n* = 23
	3.1 (0.1–35.6)	2.2 (0.9–24.7)	2.7 (1.2–10.4)	5.1 (0.1–35.6)
Median number of treatment lines to achieve first response (CR, PR), *n* (range)	*n* = 62	*n* = 26	*n* = 14	*n* = 22
	1.0 (1.0–4.0)	1.0 (1.0–3.0)	1.0 (1.0–3.0)	1.0 (1.0–4.0)
Median DOR (CR, PR), months (95% CI)	*n* = 61	*n* = 26	*n* = 12	*n* = 23
	37.0 (19.1–NR)	23.7 (13.9–NR)	29.9 (4.6–NR)	NR
Median OS, months (95% CI)				
From diagnosis of cHL	275.5 (66.2–275.5)	80.7 (41.8–NR)	275.5 (48.6–275.5)	NR
From the first relapse	87.0 (59.6–NR)	87.0 (59.6–NR)	NR	47.8 (4.2–NR)
OS rate from first relapse, % (95% CI)				
1-year	86.8 (72.9–93.9)	100.0	85.7 (53.9–96.2)	68.4 (35.9–86.8)
3-year	72.0 (55.9–83.1)	77.8 (51.1–91.0)	77.1 (44.2–92.1)	59.8 (28.5–81.0)
5-year	66.0 (49.1–785)	69.1 (39.8–86.3)	77.1 (44.2–92.1)	49.9 (20.1–74.0)
Median PFS in patients with SCT, months (95% CI)	*n* = 45	*n* = 28	*n* = 10	*n* = 7
	29.2 (14.6–48.4)	20.8 (10.8–NR)	33.3 (2.2–NR)	34.0 (0.0–NR)
PFS rate in patients with SCT, % (95% CI)				
1-year	68.9 (53.2–80.3)	64.3 (43.8–78.9)	80.0 (40.9–94.6)	71.4 (25.8–92.0)
3-year	44.4 (29.7–58.2)	42.9 (24.6–60.0)	50.0 (18.4, 75.3)	42.9 (9.8–73.4)
5-year	32.6 (19.4–46.5)	34.6 (17.7–52.3)	30.0 (7.1–57.8)	28.6 (4.1–61.2)
Median PFS in non-SCT patients, months (95% CI)				
ABVD (*n* = 41)	9.6 (2.6–29.6)	2.5 (1.3–29.0)	9.6 (1.0–NR)	11.6 (0.0–NR)
BEACOPP (*n* = 9)	2.5 (0.0–11.6)	3.0 (1.4–NR)	NR	1.7 (0.0–11.6)

*ABVD* doxorubicin bleomycin vinblastine dacarbazine, *BEACOPP* bleomycin etoposide doxorubicin cyclophosphamide vincristine procarbazine prednisone, *cHL* classical Hodgkin lymphoma, *CI* confidence interval, *CR* complete remission, *DOR* duration of response, *NR* not reached, *OS* overall survival, *PD* progressive disease, *PFS* progression-free survival, *PR* partial remission, *RRHL* relapsed/refractory classical Hodgkin lymphoma, *SCT* stem cell transplantation, *SD* stable disease.

^a^Seventy patients with an initial diagnosis of frontline cHL progressed to RRHL during the study period and were included in both groups.

^b^Unknown: there is no entry in the medical record or patient chart relating to an item as confirmed by the site.

### Patients with frontline cHL

#### Patient demographic and baseline characteristics

Patients in the frontline cHL group had a median age of 40.0 years, with a similar proportion of males and females. The most common ethnicity was White or Caucasian (20.9%); however, it was not specified in most patients (49.7%). Most patients had Ann Arbor stage III or IV disease (71.2%) at first diagnosis, with nodular sclerosis (60.5%) or mixed cellularity (23%) being the most common subtypes (Table 4). B symptoms were present in most patients (85.8%), while less than 30% had extranodal involvement or bulky disease. At baseline, approximately 1 of 3 patients had an International Prognostic Score (IPS) recorded were classified as ‘Poor’ (34.1%), ranging from 26.7% in Mexico to 54.1% in Colombia.

**Table 4:** Baseline patient characteristics, treatment patterns, and clinical outcomes in patients with cHL in Latin America: overall and by country.

Baseline characteristics	Overall (*n* = 344)^a^	Argentina (*n* = 96)	Colombia (*n* = 137)	Mexico (n = 111)
Median age, years (range)	40.0 (18.0–89.0)	37.0 (27.0–53.5)	44.0 (29.0–60.0)	36.0 (24.0–51.0)
< 60, *n* (%)	280 (81.4)	80 (83.3)	102 (74.5)	98 (88.3)
≥ 60, *n* (%)	64 (18.6)	16 (16.7)	35 (25.5)	13 (11.7)
Male, *n* (%)	171 (49.7)	50 (52.1)	66 (48.2)	55 (49.5)
Ethnicity/race, *n* (%)				
White/Caucasian	72 (20.9)	72 (75.0)	0 (0.0)	0 (0.0)
Other^b^	171 (49.7)	6 (6.3)	60 (43.8)	105 (94.6)
Ann Arbor stage at first diagnosis, *n* (%)				
IA–IIA	0 (0.0)	0 (0.0)	0 (0.0)	0 (0.0)
IIB	99 (28.8)	31 (32.3)	24 (17.5)	44 (39.6)
IIIA	29 (8.4)	14 (14.6)	8 (5.8)	7 (6.3)
IIIB	108 (31.4)	21 (21.9)	46 (33.6)	41 (36.9)
IVA	20 (5.8)	8 (8.3)	9 (6.6)	3 (2.7)
IVB	88 (25.6)	22 (22.9)	50 (36.5)	16 (14.4)
Unknown^c^	0 (0.0)	0 (0.0)	0 (0.0)	0 (0.0)
PET/PET-CT at baseline, *n* (%)	46/178 (25.8)	38/80 (47.5)	1/71 (1.4)	7/27 (25.9)
Presence of B symptoms, *n* (%)	295 (85.8)	74 (77.1)	120 (87.6)	101 (91.0)
Extranodal involvement at diagnosis, *n* (%)	95/343 (27.7)	27/96 (28.1)	42/136 (30.9)	26/111 (23.4)
Bulky disease ≥ 5 cm at diagnosis, *n* (%)	102/343 (29.7)	42/96 (43.8)	20/136 (14.7)	40/111 (36.0)
ECOG PS, *n* (%)	*n* = 190	*n* = 58	*n* = 49	*n* = 83
0	102 (53.7)	28 (48.3)	21 (42.9)	53 (63.9)
1	74 (38.9)	22 (37.9)	24 (49.0)	28 (33.7)
2	11 (5.8)	7 (12.1)	3 (6.1)	1 (1.2)
3	3 (1.6)	1 (1.7)	1 (2.0)	1 (1.2)
4	0 (0.0)	0 (0.0)	0 (0.0)	0 (0.0)
Unknown^c^	154	38	88	28
cHL histological subtype, *n* (%)				
Nodular sclerosis	208 (60.5)	68 (70.8)	68 (49.6)	72 (64.9)
Mixed cellularity	79 (23.0)	21 (21.9)	24 (17.5)	34 (30.6)
Lymphocyte-depleted	8 (2.3)	1 (1.0)	3 (2.2)	4 (3.6)
Lymphocyte-rich	6 (1.7)	2 (2.1)	3 (2.2)	1 (0.9)
Unknown^c^	43 (12.5)	4 (4.2)	39 (28.5)	0 (0.0)
IPS category, *n* (%)	*n* = 179	*n* = 82	*n* = 37	*n* = 60
Good (0–1)	34 (19.0)	17 (20.7)	1 (2.7)	16 (26.7)
Fair (2–3)	84 (46.9)	40 (48.8)	16 (43.2)	28 (46.7)
Poor (4–7)	61 (34.1)	25 (30.5)	20 (54.1)	16 (26.7)
Unknown^c^	165	14	100	51
Treatment patterns				
Frontline chemotherapy, *n* (%)	344 (100)	96 (100)	137 (100)	111 (100)
Most common regimen	ABVD 323 (93.9)	ABVD 91 (94.8)	ABVD 130 (94.9)	ABVD 102 (91.9)
Second most common regimen	CHOP 6 (1.7)	BEACOPP 4 (4.2)	‘Other’ 4 (2.9)	CHOP 6 (5.4)
Number of chemotherapy regimens, median (range)	1.0 (1.0–3.0)	1.0 (1.0–2.0)	1.0 (1.0–3.0)	1.0 (1.0–1.0)
Patients receiving PET or PET-CT scans, *n* (%)	178 (51.7%)	80 (83.3%)	71 (51.8%)	27 (24.3%)
Number of PET or PET-CT scans, median (range)	1.0 (1.0–10.0)	2.0 (1.0–10.0)	1.0 (1.0–5.0)	1.0 (1.0–3.0)
Patients receiving CT scans, *n* (%)	271 (78.8%)	68 (70.8%)	118 (86.1%)	85 (76.6%)
Number of CT scans, median (range)	2.0 (1.0–9.0)	2.0 (1.0–8.0)	2.0 (1.0–9.0)	2.0 (1.0–6.0)
Radiotherapy at frontline treatment, *n* (%)	64 (18.6)	8 (8.3)	24 (17.5)	32 (28.8)
Median number of radiotherapy treatments, *n* (range)	1.0 (1.0–2.0)	1.0 (1.0–1.0)	1.0 (1.0–2.0)	1.0 (1.0–1.0)
Clinical outcomes				
Median PFS, months (95% CI)	NR	–	–	97.6 (59.3–NR)
PFS rate, % (95% CI)				
1-year	80.0 (75.2–83.9)	76.9 (67.1–84.2)	80.4 (72.1–86.4)	82.6 (74.0–88.5)
3-year	66.9 (61.3–71.9)	58.9 (48.3–68.0)	75.4 (66.4–82.3)	65.8 (55.6–74.2)
5-year	61.6 (55.8–66.9)	55.6 (45.0–64.9)	69.9 (60.3–77.6)	58.7 (48.1–67.8)
Best clinical response following frontline treatment, *n* (%)				
CR	214 (62.2)	67 (69.8)	77 (56.2)	70 (63.1)
PR	57 (16.6)	13 (13.5)	18 (13.1)	26 (23.4)
SD	11 (3.2)	4 (4.2)	4 (2.9)	3 (2.7)
PD	30 (8.7)	8 (8.3)	10 (7.3)	12 (10.8)
Unknown^c^	32 (9.3)	4 (4.2)	28 (20.4)	0 (0.0)
Median time to response (CR, PR), months (range)	*n* = 294	*n* = 89	*n* = 99	*n* = 106
	5.1 (0.0–33.0)	3.6 (1.1–17.7)	5.6 (0.1–33)	5.5 (0–23.3)
Median number of treatment lines to achieve first response (CR, PR), *n* (range)	1.0 (1.0–4.0)	1.0 (1.0–3.0)	1.0 (1.0–4.0)	1.0 (1.0–3.0)
Median DOR (CR, PR), months (95% CI)	NR	NR	NR	88.0 (79.0–NR)
Median OS from diagnosis of cHL, months (95% CI)	NR	NR	NR	NR
OS rate from diagnosis of cHL, % (95% CI)				
1-year	95.4 (92.6–97.2)	96.9 (90.6–99.0)	92.1 (85.8–95.7)	98.1 (92.7–99.5)
3-year	85.3 (80.7–88.9)	84.0 (74.9–90.0)	88.1 (80.7–92.8)	84.0 (74.8– 90.1)
5-year	79.4 (74.2–83.7)	77.0 (66.9–84.4)	84.7 (76.3–90.3)	76.7 (66.5–84.2)

*ABVD* doxorubicin bleomycin vinblastine dacarbazine, *BEACOPP* bleomycin etoposide doxorubicin cyclophosphamide vincristine procarbazine prednisone, *cHL* classical Hodgkin lymphoma, *CHOP* cyclophosphamide doxorubicin vincristine prednisone, *CI* confidence interval, *CR* complete remission, *CT* computed tomography, *DOR* duration of response, *ECOG PS* Eastern Cooperative Oncology Group Performance Status, *IPS* International Prognostic Score, *NR* not reached, *OS* overall survival, *PD* progressive disease, *PET* positron emission tomography, *PFS* progression-free survival, *PR* partial response, *SD* stable disease.

^a^Seventy patients with an initial diagnosis of cHL progressed to RRHL during the study period.

^b^The ‘Other’ category in ‘Ethnicity/race’ combines the following categories: black or African American, American Indian or Alaska Native, Native Hawaiian or another Pacific Islander, or Other.

^c^Unknown: there is no entry in the medical record or patient chart relating to an item as confirmed by the site.

#### Treatment patterns

In the frontline cHL group, all patients received chemotherapy, the most common induction regimen being ABVD (93.9%) (Table 4). Radiotherapy as part of the induction regimen was used in 18.6% of patients and was more frequently used in Mexico than in Argentina and Colombia. PET/PET-CT scans were performed in 51.7% of patients and were more common at the end of frontline treatment (87.1%) than at baseline (25.8%). CT scans were performed in 78.8% of patients with frontline cHL, with 72.7% of these performed at baseline and 68.3% during or at the end of frontline treatment.

#### Clinical outcomes

In the frontline cHL group, the median PFS and OS were not reached. Approximately 2 in 3 patients (62.2%) achieved CR following frontline treatment (ranging from 56.2% in Colombia to 69.8% in Argentina). The 1-, 3-, and 5-year OS rates in the Latin America frontline cHL group were 95.4%, 85.3%, and 79.4%, respectively.

### AEs and deaths

At least one AE was reported in 39.0% of patients in the frontline cHL group and 51.1% of patients with RRHL. Of the 73 deaths in the frontline cHL group, 57.5% were from an HL-related event, and the cause of death was reported as ‘treatment-related’ in two patients. In the RRHL group, 34 deaths were reported, of which 64.7% were from an HL-related event, and the cause of death was reported as ‘treatment-related’ in one patient.

## Discussion

The B-HOLISTIC Latin America subgroup analysis represents a comprehensive analysis of real-world data on treatment patterns and clinical outcomes in patients with HL observed for over 3 years. Overall, the treatment patterns observed in patients with frontline cHL and RRHL in the Latin America subgroup were generally aligned with the European Society for Medical Oncology and the National Comprehensive Cancer Network guideline recommendations at the time of the study^4,5^. However, clinical outcomes were suboptimal and consistent with previous findings^14,16,21^. Given the limited real-world evidence in HL in Latin America, these findings may offer valuable insights into HL treatment and outcomes and help serve as a reference for future studies in HL in this region.

Demographic and baseline data were in line with expectations for this region^26,27^ with the exception of similar proportions of male and female patients, in contrast to other observational studies on HL^10,28–30^. The treatment patterns and the choice of preferred regimen for frontline (ABVD) and salvage treatments (ICE and ESHAP) across the three countries were consistent with guideline recommendations^4,5^ and clinical practices at the time of the study. Since the guidelines do not recommend any specific chemotherapy regimens, these findings can help guide the choice of regimens in these patients based on their baseline clinical characteristics and preferences. ABVD was used as the salvage regimen in four patients. The rationale for using ABVD as salvage therapy was not documented. Although this is not standard practice, these cases were classified as late relapses (occurring more than 18 months after frontline therapy), which may have influenced the treatment decision. The use of novel agents like brentuximab vedotin and rituximab was noted only in later lines of treatment or post-SCT settings. Real-world evidence suggests that despite advance in treatment options, LMICs rely on conventional salvage regimens for RRHL probably owing to access, cost, slower uptake of novel targeted therapies, or a lack of recommendations on their use at the time of the study^19,31,32^. SCT was performed in two-thirds of eligible patients with RRHL in this region; there were notable country-level differences in patients undergoing SCT. In the frontline cHL group, the use of baseline PET/PET-CT scans was limited (25.8%). A similar trend was observed in a Brazilian Hodgkin Lymphoma Registry study (2009–2018), which reported a utilization rate of 24% for PET-CT in staging^16^. Although PET/PET-CT scans have become standard practice for staging cHL, their use was less common between 2010 and 2013 and it remains uncommon in LMICs, including those in Latin America^14,31,33^. Factors such as the high cost of PET/PET-CT and the scarcity of available scanners likely restricted its broader adoption. However, there have been reports of an increasing trend in the use of PET-CT–guided staging in recent years^16^.

Median PFS in the RRHL group was 20.2 months, longer than that reported in the B-HOLISTIC primary study (13.2 months)^25^; however, it was suboptimal compared with reports from developed regions^28,34–36^. Median PFS was better in patients undergoing SCT than those without SCT. Factors such as limited access to and uptake of novel therapies and the high prevalence of primary refractory disease (over 1 in 3 patients) likely contributed to the observed low PFS rate^14^. The pre-SCT CR rates, an important factor for post-SCT outcomes^37,38^, were low (50%) in the Latin America subgroup, which may also have influenced the feasibility to perform SCT and the suboptimal post-SCT outcomes.

The variation in treatment choices, inconsistent SCT rates, limited use of PET/CT, and suboptimal clinical outcomes in the B-HOLISTIC Latin America subgroup reflect broader healthcare disparities. These stem from differences in healthcare policies, heterogenous patient populations, and the slow adoption of contemporary diagnostic techniques and novel and effective cancer treatments. Additionally, limited or inequitable access to treatments, alongside affordability challenges, further contribute to the variability in clinical outcomes across Latin American countries^13,39,40^. These challenges in HL treatment persist in the region. While developed countries focus on optimizing existing treatment regimens, resource-limited regions benefit more from efforts to improve treatment outcomes. Strategies to improve access to novel treatments and introducing them earlier in treatment, improving progression to SCT, and adapting international guidelines to local clinical settings could significantly improve clinical outcomes in HL in Latin America.

This study is one of the first large observational studies to examine treatment patterns and clinical outcomes among patients with HL in Latin America. However, several limitations of the B-HOLISTIC study, which have been discussed previously, may affect the interpretation of the data^25^. The Latin America subgroup included patients only from Argentina, Colombia, and Mexico—predominantly from specialized treatment centers—with a small sample size, and all patients from Mexico were enrolled from a single center, which may limit the representativeness of the subgroup and the generalizability of the study findings. Additionally, data from Latin American national registries, including the Brazilian Hodgkin Lymphoma Registry, were not incorporated into this study. The study did not differentiate clearly between Hispanic and Latino ethnicities, which may affect the interpretation of the impact of race on the study findings. Further, including patients with cHL who progressed to RRHL in both frontline cHL and RRHL groups may have impacted the overall results. Finally, statistical or indirect comparisons with global or other regional subanalyses were not feasible due to the lack of harmonized datasets across regions. Future studies should build on our findings by integrating harmonized datasets and conducting more rigorous comparative analyses to further enhance our understanding of regional variations in disease presentation and outcomes.

In conclusion, the B-HOLISTIC study represents a significant advancement in understanding treatment patterns and long-term clinical outcomes in patients with HL in Latin America. The results reveal suboptimal outcomes and limited access to novel therapies, SCT, and PET/PET-CT scans in these regions, when compared with that reported in Europe and North America. To improve patient outcomes, it is crucial to enhance healthcare access, promote timely adoption of novel treatments, and consider tailored approaches that address the unique challenges faced in resource-limited settings. By implementing these measures, we can strive for better clinical outcomes and bridge the gap in HL care in Latin America.

## Electronic supplementary material

Below is the link to the electronic supplementary material.

Supplementary Material 1

## Data Availability

The datasets, including the redacted study protocol, redacted statistical analysis plan, and individual participant’s data supporting the results reported in this article, will be made available within 3 months from the initial request to researchers who provide a methodologically sound proposal. The data will be provided after its de-identification, in compliance with applicable privacy laws, data protection, and requirements for consent and anonymization. You may address data request to the corresponding author, Dr. Amado J Karduss, amaka962@gmail.com.
