# Supplementary material for: Subgroup analysis of treatment pathways and clinical outcomes in Hodgkin lymphoma in Latin America from the retrospective B-HOLISTIC study
Source: Sci Rep. 2025 Jul 7;15:24197. doi: 10.1038/s41598-025-07704-0 (PMC12234794; doi:10.1038/s41598-025-07704-0)
Supplement: Supplementary file 1 — Supplementary Material 1 [file 41598_2025_7704_MOESM1_ESM.docx]

# Supplementary material

**Subgroup analysis of treatment pathways and clinical outcomes in Hodgkin lymphoma in Latin America from the retrospective B-HOLISTIC study**

Alvaro Hernandez-Caballero^1^, Ruben Salazar^2^, Marta Zerga^3^, Silvia Rivas-Vera^4^, Zhongwen Huang^5^, Amado Karduss^6^

^1^Unidad Medica de Alta Hospital de Especíalídades Dr. Antonio Fraga Mouret Centro Medico Nacional la Raza, México City, Mexico

^2^Clínica de Oncología Astorga, Medellín, Colombia

^3^Instituto de Oncología Angel H. Roffo, Buenos Aires, Argentina

^4^Department of Hematology, Instituto Nacional de Cancerología México, México City, Mexico

^5^Takeda R&D Data Science Institute, Takeda Pharmaceuticals International Co., Cambridge, USA

^6^Instituto de Cancerología Las Americas AUNA, Medellín, Colombia

**Corresponding author:**

Dr. Amado Karduss,

Instituto de Cancerología Las Americas,

Medellín, Colombia.

Email: [amaka962@gmail.com](mailto:amaka962@gmail.com); [amaka@une.net.co](mailto:amaka@une.net.co)

#### **Supplementary Table 1** Latin-America B-HOLISTIC study sites and principal investigators

| Participating center | Country | Principal investigator | |
| --- | --- | --- | --- |
| Hospital Italiano de Buenos Aires | Argentina | Dorotea | Fantl |
| Instituto de Oncología Ángel Roffo | Argentina | Marta | Zerga |
| Academia Nacional de Medicina de Buenos Aires | Argentina | Miguel | González |
| Hospital Privado Centro Medico de Cordoba S.A. | Argentina | Luciana | Guanchiale |
| Hospital Universitario Austral | Argentina | María Marta | Rivas |
| Hospital Británico | Argentina | Claudia | Shanley |
| Instituto Medico Especializado Alexander Fleming | Argentina | Adriana | Vitriu |
| Centro de Investigación y Prevención Cardiovascular | Argentina | Adrián | Huñis |
| Instituto de Cancerología S.A. | Colombia | Amado | Karduss |
| Sociedad de Cirugía de Bogotá Hospital de San José | Colombia | Virginia | Abello Polo |
| Hospital Pablo Tobón Uribe | Colombia | Kenny | Galvez |
| Fundación Hospitalaria San Vicente de Paul | Colombia | Jorge | Cuervo-Sierra |
| Clínica De Oncología Astorga | Colombia | Rubén | Salazar |
| Fundación Oftalmológica de Santander – FOSCAL | Colombia | Luis | Salazar |
| Hospital Universitario Mayor – Méderi | Colombia | Jair | Figueroa |
| Fundación Cardioinfantil – Instituto de Cardiología | Colombia | Andres Armando | Borda Molina |
| Centro de Investigacíon Farmacéutica Especializada de Occidente S.C. | Mexico | Juan Antonio | Flores Jiménez |
| aUnidad Medica de Alta Hospital de Especíalídades Dr. Antonio Fraga Mouret Centro Medico Nacional la Raza | Mexico | Álvaro | Hernández |
| Antiguo Hospital Civil de Guadalajara Fray Antonio Alcalde | Mexico | Francisco Javier | Ramírez Godinez |
